# Cost-effectiveness of abemaciclib plus endocrine therapy in high-risk HR+/HER2–early breast cancer in China

**DOI:** 10.1186/s12962-023-00499-9

**Published:** 2023-11-27

**Authors:** Qiran Wei, YuTing Xu, Wei Liu, Xin Guan

**Affiliations:** 1 School of International Pharmaceutical Business, China Pharmaceutical University, Nanjing, 211198 Jiangsu China; 2 Center for Pharmacoeconomics and Outcomes Research of China Pharmaceutical University, Nanjing, 211198 Jiangsu China

**Keywords:** Cost-effectiveness, Abemaciclib, Early breast cancer, High risk, Adjuvant treatment

## Abstract

**Objective:**

The aim of this article is to evaluate the cost-effectiveness of abemaciclib plus endocrine therapy (ABE + ET) vs. ET as adjuvant treatment for high-risk hormone receptor-positive and human epidermal growth factor receptor 2-negative (HR+/HER2–) early breast cancer in China.

**Methods:**

From the perspective of the Chinese health care system, a 5-state Markov model was developed with a lifetime horizon. Data of the monarchE phase III clinical trial were used to model the invasive disease-free survival (iDFS) and standard parameters models were used for data extrapolation. Costs were obtained from national data sources, expert opinions and published literature using 2023 US dollars and discounted by 5%. The results were evaluated in terms of life-years (LYs) and quality-adjusted life-years (QALYs). Sensitivity analyses and scenario analyses were performed to test the robustness of the basic results.

**Results:**

In the base-case analysis result, the model projected improved outcomes (by 0.65 LYs and 0.72 QALYs) and increased costs (by $16,057.72) for incremental cost-effectiveness ratios (ICERs) of $24,841/LY and $22,385/QALY for ABE + ET vs. ET patients. The results in scenario analysis estimated the ICERs of ABE + ET treatment to be $16,959/LY and $15,264/QALY in a mixture cure model, and $13,560/LY and $12,191/QALY in a non-mixture cure model. The model was sensitive to outcome discount rate and utility of iDFS.

**Conclusion:**

ABE + ET might not have an economic advantage over ET at a willingness-to-pay (WTP) threshold of one time the per capita GDP in China, but was expected to be more cost-effective at a WTP threshold of three times the per capita GDP. Further analysis will be conducted once data from longer-term studies become available.

**Supplementary Information:**

The online version contains supplementary material available at 10.1186/s12962-023-00499-9.

## Introduction

Breast cancer is a prevalent cancer worldwide, with over 2.26 million new cases reported in 2020. In China, there were approximately 420,000 new diagnoses of breast cancer in the same year, with a 5-year prevalence rate of 197.0 per 100,000 people [1]. Breast cancer is also the leading cause of cancer-related deaths among Chinese women, resulting in approximately 120,000 deaths annually [1, 2]. Furthermore, there has been a noticeable increase in the incidence of breast cancer in China [3], and the growth rate exceeds the global average [4].

Breast cancer can be classified into various subtypes based on the expression of human epidermal growth factor receptor (HER2) and hormone receptor (HR). HR+/HER2− subtype makes up over 70% of all primary breast cancers [5]. 90% of breast cancer patients are diagnosed in the early stages [6], and 20% of these early-stage HR+/HER2− patients face the risk of recurrence or progression to incurable metastatic cancer within the first 10 years [7]. Current treatment options for early HR+/HER2− breast cancer (eBC) include surgery, radiotherapy, chemotherapy, and adjuvant endocrine therapy (ET), with aromatase inhibitors and tamoxifen being the standard form of ET [8]. However, there is still a significant unmet need for patients at high risk of recurrence, as the current treatments have limited effectiveness [9, 10].

In recent years, the addition of a cyclin-dependent kinase (CDK4/6) inhibitor to ET has become a research focus. These inhibitors work by blocking the progression of cells from G1 phase to S phase, thus inhibiting the DNA synthesis and proliferation of tumor cells [11]. Three CDK4/6 inhibitors (palbociclib, ribociclib, and abemaciclib) have been approved by both the US Food and Drug Administration and the European Medicines Agency, with abemaciclib (ABE) being one of them [11, 12]. ABE is a selective CDK4 and CDK6 small molecule inhibitor administered continuously on a twice-daily schedule [13]. It has a higher selectivity against CDK4 compared to CDK6 among all three CDK4/6 inhibitors [14], and has been recommended for adjuvant treatment of high-risk eBC [15] following the results of the monarchE trial (Clinicaltrials.gov registration: NCT03155997) [16]. The monarchE trial was an open-label, global, randomized, phase III study that evaluated the efficacy of ABE in combination with endocrine therapy (ABE + ET) versus ET alone in HR+/HER2−, node-positive, high-risk eBC patients. High risk was defined based on axillary lymph node status, primary invasive tumor size, tumor histological grade, and Ki-67 index (cut-off of 20%). Ki-67 is a proliferative marker, and high Ki-67 expression is associated with worse prognosis in the eBC population [17]. The trial showed a significant reduction in the risk of recurrence for high-risk eBC patients receiving ABE + ET (hazard ratio, 0.71; 95% CI 0.58–0.87; p < 0.001). However, grade ≥ 3 adverse effects occurred in 50% of patients receiving ABE + ET, compared with 15% of patients receiving ET alone [18].

In 2022, ABE + ET was approved by the Chinese National Medical Products Administration for the adjuvant therapy of adult patients with HR+, HER2−, positive lymph nodes, high recurrence risk and Ki-67 ≥ 20% eBC. When considering a new treatment, the potential benefits, such as improved progression-free survival, must be balanced against potential harms, such as therapeutic toxicity and increased costs. Economic evaluations, which compare the economic and health outcomes of new interventions, are crucial for healthcare providers and policy makers. Therefore, the purpose of this article was to assess the cost-effectiveness of ABE + ET for high-risk HR+/HER2− eBC patients in China. Additionally, the study conducted subgroup analysis using the population parameters of Ki-67 ≥ 20% from the monarchE trial, in accordance with the approved indication for ABE.

## Methods

### Model overview

**Fig. 1:**
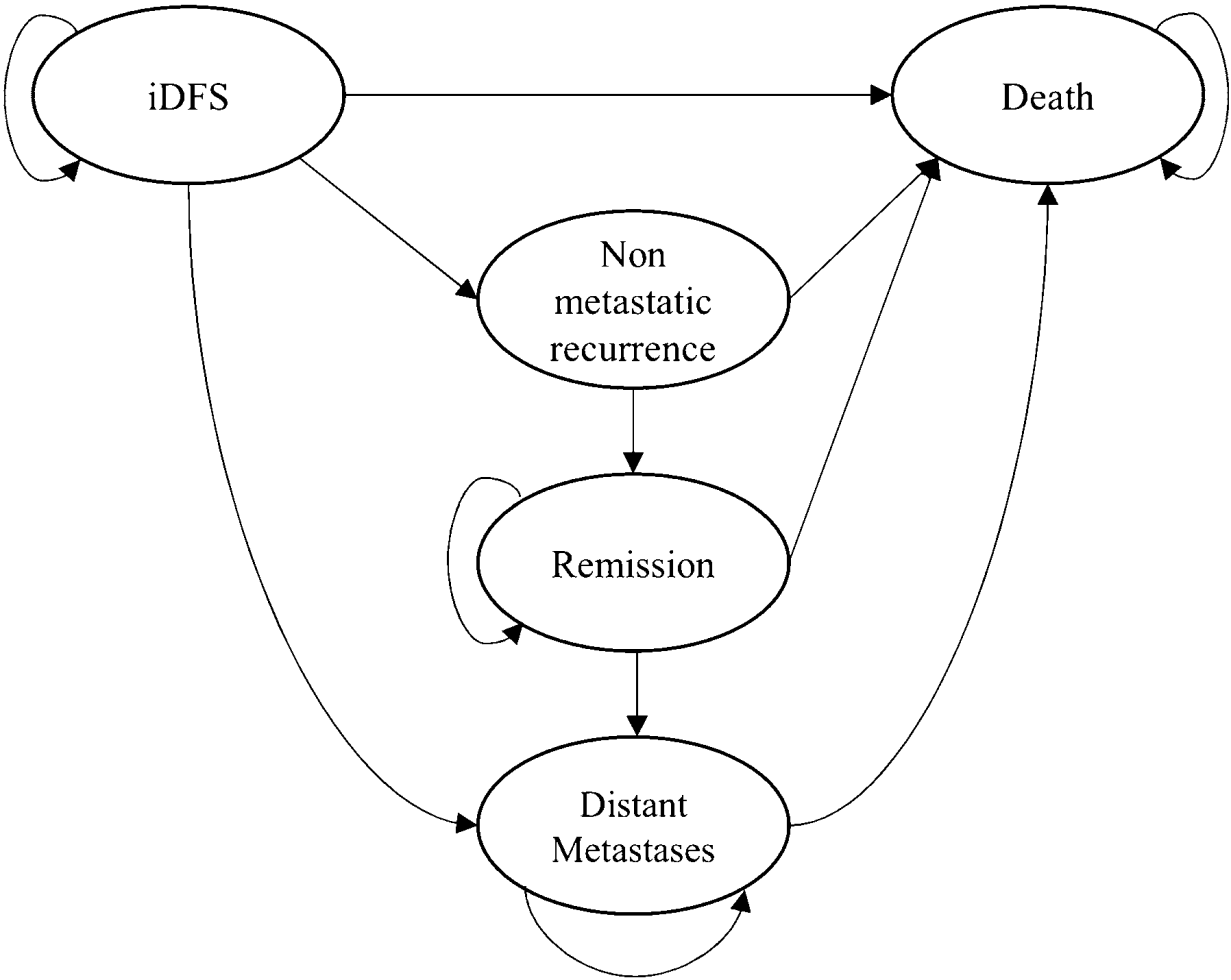
Model structure iDFS, invasive disease-free survival

A probabilistic Markov model was designed in Microsoft Excel to estimate the lifetime costs and health outcomes for the adjuvant treatment of patients with high-risk HR+/HER2− eBC. As shown in Fig. 1, the model included five health states: invasive disease-free survival (iDFS), nonmetastatic recurrence (including locoregional recurrence and contralateral breast cancer), remission, metastatic recurrence, and death. All patients received their assigned adjuvant therapy and are considered to be in the iDFS health state if they neither died due to natural causes nor experienced a metastatic or nonmetastatic recurrence. The nonmetastatic recurrence health state is a temporary state where patients remain for 5 years if they don’t experience a death event in this period. The duration of the tunnel state was chosen to be 5 years as it is expected that patients will undergo another round of adjuvant therapy. After the 5 year period in nonmetastatic recurrence health state, patients transition to remission and stop receiving treatment. The assumption is that in the iDFS, nonmetastatic recurrence, and remission states, further progression of the disease will lead to metastasis rather than death from the disease. It is also assumed that 95% of patients with event-free survival for over 10 years will be considered cured. Research has shown that after 10 years of adjuvant therapy, the risk of disease progression, including death, in eBC patients is comparable to the risk of death in the general population [19].

The model was simulated from the perspective of the Chinese health care system. Each cycle lasted 1 month, which was consistent with the dose schedule in the monarchE trial. The results were evaluated in terms of life-years (LYs) and quality-adjusted life-years (QALYs). Both costs and health outcomes were discounted at a rate of 5%.

### Patient population

The target population of this study was consistent with the monarchE trial, and they received one of the following two treatments at the beginning of the model.

1. ABE + ET group: The patients received ABE 150 mg twice daily for 2 years. The selection of a two-year treatment duration for exposing patients to ABE is supported by consultations with clinical experts, who affirmed its alignment with real-world practices. In addition, letrozole, the most commonly used endocrine drug in monarchE trial [20], was selected as the standard ET. The patients received letrozole 2.5 mg daily for 5 years.
2. ET group: The patients received letrozole 2.5 mg daily for 5 years.

All patients underwent treatment until they experienced disease progression or death. The age of treatment onset was 52.2 years for the ABE + ET group and 52.1 years for the ET group.

### Clinical efficacy

**Fig. 2:**
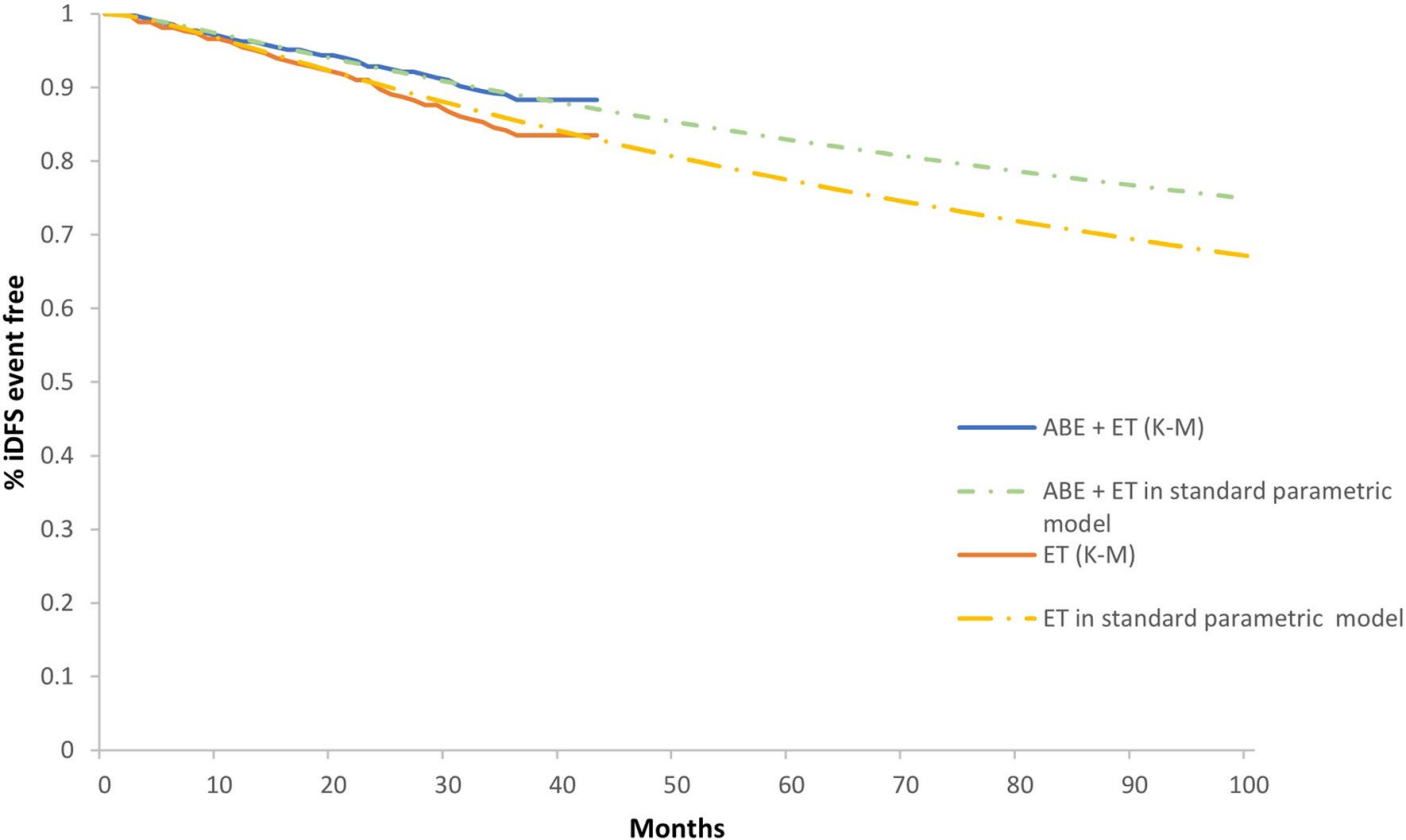
KM and parametric survival distributions for iDFS iDFS, invasive disease-free survival; ABE + ET: abemaciclib + endocrine therapy; ET: endocrine therapy

The survival probabilities for disease-free state in the two strategies were based on the monarchE trial. Extrapolation was required in the cost-effectiveness analysis due to the limited follow-up time of the Kaplan–Meier (KM) curves in clinical trials. A standard parametric model was fitted using Exponential, Generalized Gamma, Weibull, Gompertz, Loglogistic, Lognormal distributions to extrapolate the probability of iDFS. The lowest Akaike information criterion (AIC) values were selected as indicators of goodness-of-fit, and a visual examination was performed to verify if the distribution adequately fit the KM curve. The pseudo-individual patient data (IPD) were extracted with Engauge Digitizer software from the clinical trial and were then reconstructed and fitted by the standard parametric models using R 3.6.0 [21]. For the iDFS of both groups, the preferred distribution was Lognormal, as it demonstrated the best fit to survival based on AIC, which was presented in Addoitional file 1: Table S1 and S2. KM and parametric survival distributions for iDFS used in the model were shown in Fig. 2.

From randomization to 36 months, the recurrence rate was maintained at a high level in the monarchE trial, and a clear change in the incidence of events was observed between 36 and 45 months. This trend has been incorporated into the model by assuming that from 36 months onwards, the proportion of patients being cured increases linearly over time, reaching 95% at 120 months. This 95% cure rate was determined based on a study by Takeuchi et al. [22], which analyzed the recurrence of 1,114 breast cancer patients following surgery. The study found that only 1.08% of patients would relapse 10 years later. When incorporating this 95% cure rate in the model, the results showed that 1.17% of patients would relapse after 10 years of treatment in ET group, which aligned with previous research.

The proportions of breast cancer events that would be nonmetastatic recurrence or metastatic recurrence were based on the pooled distribution in the ATAC trial [23]. The probabilities of distant disease after local or regional recurrence were derived from a prospective study [24]. The rates of distant metastases were assumed to be equivalent in both groups.

Age-specific background mortality rates were obtained from China Population Census Yearbook [25]. We estimated a monthly probability of death among women with distant metastases to be 0.0248 based on survival data from the Letrozole P025 trial, which evaluated the efficacy of first-line letrozole treatment in patients with advanced disease [26].

### Cost

We estimated the costs in 2023 US dollars, with an exchange rate of $1 = ￥6.81 (February 12st, 2023) inflated using the Medical Consumer Price Index (http://www.stats.gov.cn/tjsj./ndsj/). From the perspective of the Chinese health care system, only direct medical care costs were considered. These included drugs, follow-up, administration, end of life, subsequent treatment, and adverse events (AEs) costs.

The treatment costs per cycle were calculated by multiplying the unit costs of the drugs by the dosing schedules for one cycle and the costs of drugs were based on the median price of the bid-winning products on China Drug Bidding Database (https://www.menet.com.cn/). We captured the proportion of ET and chemotherapy regimens in the nonmetastatic recurrence state from expert opinions by administering a questionnaire to clinical experts. For patients with distant metastases, the proportions of treatment were obtained from the real-world study of HER2−/HR+ advanced breast cancer in China [27]. In this study, most patients relapsed after receiving curative surgery and adjuvant therapy, which is consistent with our research background. To estimate the dosages of chemotherapy, a body weight of 60 kg and a body surface area of 1.60 m^2^ were assumed. We employed a cost per unit of drug approach to account for unused drug waste in the model. The assumption was that all patients received the full prescribed doses of their assigned treatment with a consistent dose intensity. The follow-up and administration costs were based on the median price of the health care documents. The costs for end-of life care were obtained from the published literature [28]. The potential impact of grade 3 or 4 AEs (≥ 5%) during the adjuvant therapy was considered in the model. The cost of AEs was calculated based on expert opinions.

### Outcome measures

QALYs were calculated by multiplying the number of LYs by the health utility. Health utility reflects the patient's quality of life, with a value of 0 indicating death and 1 indicating perfect health. Since there was limited information on quality of life collected in the monarchE trial, the utilities for health states were obtained from previous studies on breast cancer [23, 29, 30]. The disutility associated with the main adverse events were also considered [31, 32].

### Sensitivity analysis

A one-way sensitivity analysis was conducted to identify the parameters that had the greatest impact on the model. The potential impact of reducing the price of ABE was evaluated by using the base value and a 0.3 reduction of the base value. We used the minimum and maximum values of letrozole in a third-party database as the lower and upper limits. We also used discount rates of 0% and 8% as the upper and lower bounds. The upper and lower limits for the remaining input parameters were defined using published literature and 95% confidence intervals when possible, and by using plausible variations of 10% for the risk of AEs and 20% for other parameters around the base-case values. The results were presented in a tornado diagram.

A probability sensitivity analysis (PSA) using a Monte Carlo simulation was performed to evaluate the impact of uncertainty on the key parameters of the model on the incremental cost-effectiveness ratios (ICERs). Distributions for each parameter were modeled using the PSA, with gamma distributions for all costs and beta distributions for utilities and risk of AEs. The probabilistic sensitivity analysis was based on 1,000 samples, and the results were presented as a cost-effectiveness scatterplot and acceptability curve.

The parameters included in one-way sensitivity analysis and PSA, along with their variations, were shown in Table 1.

**Table 1:** Key inputs for the Markov model

Model inputs	Value	Distribution	Low	High	Source
Direct costs per cycle					
Abemaciclib	748.90	GAMMA	524.23	748.90	MENET
Letrozole	11.66	GAMMA	8.87	112.14	MENET
Follow-up visit for 1 to 2 years	125.42	GAMMA	100.34	150.51	Health care document
Follow-up visit for 3 to 5 years	73.16	GAMMA	58.53	87.80	Health care document
Follow-up visit after 5 years	24.39	GAMMA	19.51	29.51	Health care document
Administration	72.64	GAMMA	58.11	87.17	Health care document
Treatment for nonmetastatic recurrences	476.80	GAMMA	381.44	572.15	[27], MENET
Treatment for metastatic recurrence	444.00	GAMMA	355.20	532.80	[27], MENET
End-of-life care	2,689.86	GAMMA	1,596.00	2,394.00	[28]
Costs of AEs per cycle					
Diarrhea	0.42	GAMMA	0.33	0.50	Expert opinion, MENET
Neutropenia	8.61	GAMMA	6.89	10.34	Expert opinion, MENET
Lymphopenia	3.57	GAMMA	2.85	4.28	Expert opinion, MENET
Risk of AEs in ABE + ET					
Diarrhea	0.078	BETA	0.070	0.086	monarchE trial
Neutropenia	0.196	BETA	0.176	0.216	monarchE trial
Lymphopenia	0.054	BETA	0.049	0.059	monarchE trial
Health state utility (per year)					
iDFS	0.965	BETA	0.744	0.980	[23, 41, 42]
Nonmetastatic	0.766	BETA	0.725	0.780	[19, 23, 42]
Remission	0.850	BETA	0.700	0.850	[19, 43]
Distant metastases	0.642	BETA	0.615	0.690	[19, 23, 41]
Disutilities of AEs (per year)					
Diarrhea	0.103	BETA	0.124	0.082	[32]
Neutropenia	0.090	BETA	0.108	0.072	[31]
Lymphopenia	0.090	BETA	0.108	0.072	[31]
AEs duration (days)					
Diarrhea	2	CONSTANT	/	/	[44]
Neutropenia	2	CONSTANT	/	/	[31]
Lymphopenia	2	CONSTANT	/	/	[31]
Cost discount rate	5%	CONSTANT	0%	8%	[45]
Outcome discount rate	5%	CONSTANT	0%	8%	[45]

Experts here refer to two clinical oncologist experts in China with more than 10 years of clinical experience in the treatment of breast cancer

AE: adverse event; ABE + ET: abemaciclib + endocrine therapy; iDFS: invasive disease-free survival

### Scenario analysis

An alternative time-horizon (simulated until the expected life expectancy in China [25]) was tested to see how costs and benefits would be affected by a shorter time-horizon. In addition, we found the KM curve showed an obvious plateau after 36 months. Therefore, we also used cure models (both mixture cure model and non-mixture cure model) [33] to estimate the robustness of the model. For the iDFS of both models, the preferred distribution was Lognormal based on its good statistical and visual fit (Additional file 1: Table S1 and S2).

### Subgroup analysis

The key population parameters of the monarchE trial with Ki-67 ≥ 20% were used for subgroup analysis. The standard parametric model was used for both groups in subgroup analysis. The preferred distribution was Exponential in the ABE + ET group and Weibull in the ET group according to the AIC (Additional file 1: Table S3 and S4).

## Results

### Base-case results

The projected mean outcomes were better for the ABE + ET group (13.80 LYs and 13.11 QALYs) compared with the ET group (13.15 LYs and 12.39 QALYs). The projected mean costs were also higher for the ABE + ET group ($29,049.28) compared with the ET group ($12,991.56). Thus, the ICERs comparing the ABE + ET group to the ET group were $24,841/LY and $22,385/QALY (Table 2).

**Table 2:** Base case and scenario analysis results

Model	Results	ABE + ET	ET	Difference
Base-case analysis	LYs			
	LYs in iDFS	12.90	11.84	1.06
	LYs in nonmetastatic recurrence	0.27	0.39	− 0.12
	LYs in remission	0.25	0.36	− 0.11
	LYs in distant metastases	0.39	0.56	− 0.18
	Total LYs	13.80	13.15	0.65
	QALYs			
	QALYs in iDFS	12.44	11.43	1.02
	QALYs in nonmetastatic recurrence	0.20	0.30	− 0.09
	QALYs in remission	0.21	0.31	− 0.10
	QALYs in distant metastases	0.25	0.36	− 0.11
	Total QALYs	13.11	12.39	0.72
	Costs ($)			
	Costs in iDFS	24,213.41	5964.31	18,249.10
	Costs in nonmetastatic recurrence	1,927.17	2800.09	− 872.92
	Costs in remission	24.04	34.53	− 10.48
	Costs in distant metastases	2,573.46	3739.96	− 1166.50
	Costs in end-of-life care	311.19	452.68	− 141.49
	Total Costs	29,049.28	12,991.56	16,057.72
	ICER ($/LY)	24,841		
	ICER ($/QALY)	22,385		
Scenario 1 (simulated until the expected life expectancy)	LYs			
	LYs in iDFS	11.65	10.72	0.93
	LYs in nonmetastatic recurrence	0.27	0.39	− 0.12
	LYs in remission	0.24	0.35	− 0.11
	LYs in distant metastases	0.38	0.55	− 0.17
	Total LYs	12.54	12.01	0.52
	QALYs			
	QALYs in iDFS	11.24	10.35	0.89
	QALYs in nonmetastatic recurrence	0.20	0.30	− 0.09
	QALYs in remission	0.21	0.30	− 0.09
	QALYs in distant metastases	0.24	0.35	− 0.11
	Total QALYs	11.89	11.30	0.60
	Costs ($)			
	Costs in iDFS	24,213.41	5964.31	18,249.10
	Costs in nonmetastatic recurrence	1927.17	2799.89	− 872.72
	Costs in remission	24.04	34.53	− 10.48
	Costs in distant metastases	2532.04	3677.10	− 1145.07
	Costs in end-of-life care	305.81	444.51	− 138.70
	Total Costs	29,002.47	12,920.34	16,082.13
	ICER ($/LY)	30,741		
	ICER ($/QALY)	26,945		
Scenario 2 (mixture cure model)	LYs			
	LYs in iDFS	13.47	11.99	1.48
	LYs in nonmetastatic recurrence	0.20	0.37	− 0.17
	LYs in remission	0.19	0.35	− 0.16
	LYs in distant metastases	0.29	0.54	− 0.25
	Total LYs	14.14	13.24	0.90
	QALYs			
	QALYs in iDFS	12.99	11.57	1.43
	QALYs in nonmetastatic recurrence	0.15	0.29	− 0.13
	QALYs in remission	0.16	0.29	− 0.13
	QALYs in distant metastases	0.19	0.34	− 0.16
	Total QALYs	13.49	12.49	1.00
	Costs ($)			
	Costs in iDFS	24,346.98	6005.42	18,341.56
	Costs in nonmetastatic recurrence	1462.81	2678.69	− 1215.89
	Costs in remission	20.69	32.74	− 12.05
	Costs in distant metastases	1951.64	3578.09	− 1626.45
	Costs in end-of-life care	234.06	433.32	− 199.26
	Total Costs	28,016.18	12,728.27	15,287.91
	ICER ($/LY)	16,959		
	ICER ($/QALY)	15,264		
Scenario 3 (non-mixture cure mode)	LYs			
	LYs in iDFS	13.44	11.66	1.78
	LYs in nonmetastatic recurrence	0.20	0.41	− 0.21
	LYs in remission	0.19	0.38	− 0.19
	LYs in distant metastases	0.29	0.59	− 0.30
	Total LYs	14.13	13.05	1.08
	QALYs			
	QALYs in iDFS	12.97	11.25	1.72
	QALYs in nonmetastatic recurrence	0.16	0.31	− 0.16
	QALYs in remission	0.16	0.32	− 0.16
	QALYs in distant metastases	0.19	0.38	− 0.19
	Total QALYs	13.48	12.27	1.20
	Costs ($)			
	Costs in iDFS	24,322.64	5968.54	18,354.10
	Costs in nonmetastatic recurrence	1484.96	2948.07	− 1463.11
	Costs in remission	21.15	33.86	− 12.71
	Costs in distant metastases	1981.09	3939.30	− 1958.21
	Costs in end-of-life care	237.48	478.88	− 241.40
	Total Costs	28,047.32	13,368.65	14,678.67
	ICER ($/LY)	13,560		
	ICER ($/QALY)	12,191		

ABE + ET: abemaciclib + endocrine therapy; ET: endocrine therapy; LY: life-year; ICER: incremental cost-effectiveness ratio; QALY: quality-adjusted life-year

### Sensitivity analysis

**Fig. 3:**
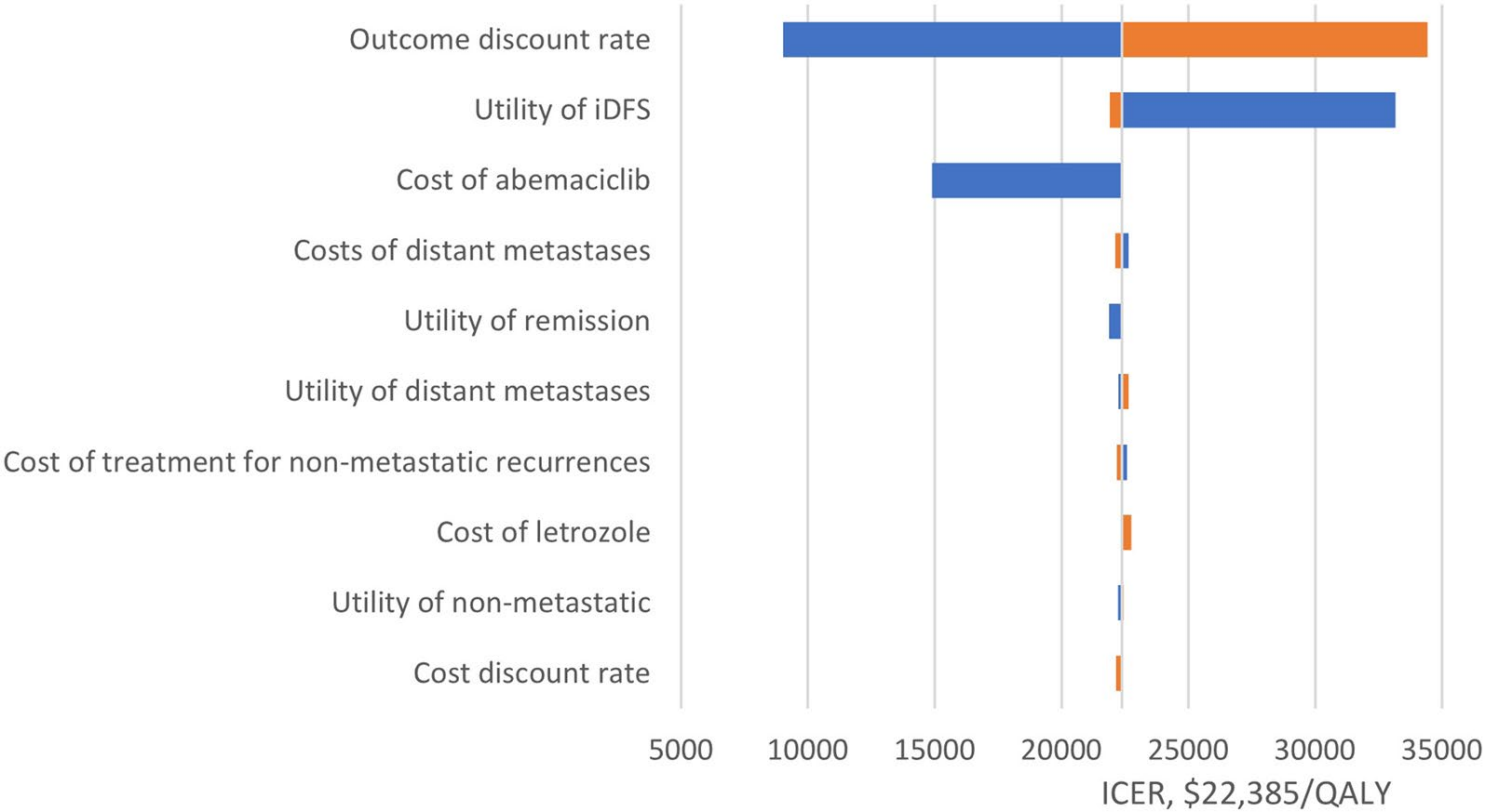
One-way sensitivity analysis iDFS, invasive disease-free survival; ICER: incremental cost-effectiveness ratio

The outcome discount rate and utility of iDFS had the greatest impact on the ICERs in all models (Fig. 3). Other parameters, such as cost of ABE, had relatively little impact on the ICERs. The range of the one-way sensitivity analysis was from $9,028/QALY to $34,431/QALY.

**Fig. 4:**
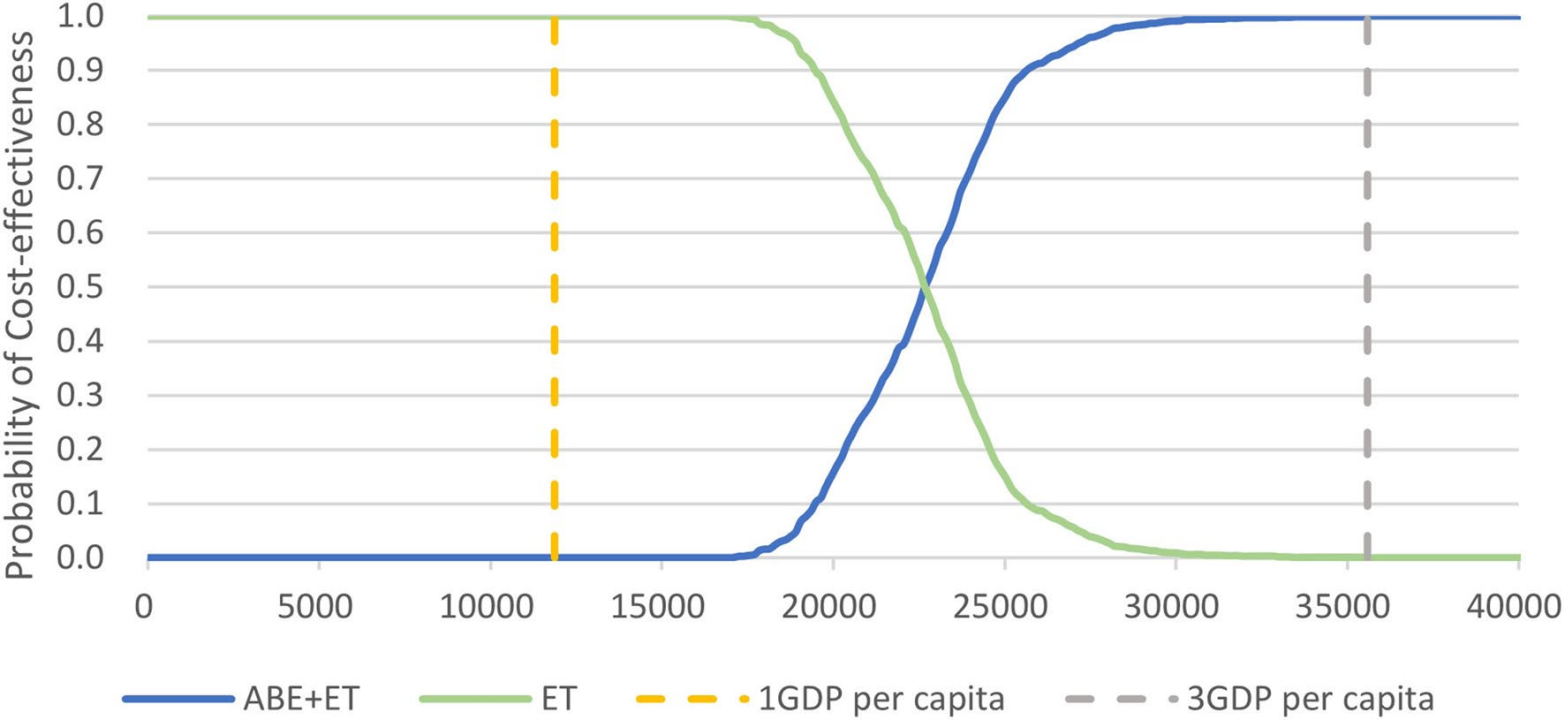
Cost-effectiveness acceptability curves at different thresholds of willingness to pay ABE + ET: abemaciclib + endocrine therapy; ET: endocrine therapy

A scatterplot in the cost-effectiveness plane demonstrates that the majority of the 1,000 iteration results from the probabilistic sensitivity analysis landed in the northeast quadrant, indicating that ABE + ET was more effective but also came with an increasing cost (Additional file 1: Figure S1). The study applied the WHO criteria for determining cost-effectiveness, which was based on a willingness-to-pay (WTP) threshold of 1–3 times the GDP per capita per QALY [34]. The cost-effectiveness acceptability curves (Fig. 4) indicated that ET alone was cost-effective at a WTP threshold of $11,864/QALY (1 GDP per capita), and the probability of ABE + ET being cost effective was 100% at a WTP threshold of $35,594/QALY (3 GDP per capital).

### Scenario analysis

As the time horizon shortened, the ICERs of ABE + ET increased. In a context where the average life expectancy of the Chinese population is 78 years, a 26-year time horizon resulted in the ICERs of $30,741/LY and $26,945/QALY.

The use of the cure models brought more survival benefits to patients than the standard parametric model. If the mixture cure model was considered, ABE + ET treatment provided an additional 1.00 QALYs and 0.90 overall LYs in comparison with ET with the ICERs of $16,959/LY and $15,264/QALY. If the non-mixture cure model was considered, ABE + ET treatment provided an additional 1.20 QALYs and 1.08 overall LYs in comparison with ET. The ICERs in the Markov model were $13,560/LY and $12,191/QALY (Table 2).

### Subgroup analysis

The results of the subgroup analysis found that the total cost of the ABE + ET group was $44,586.45 compared with $16,709.21 for the ET group. When considering the outcome, the ABE + ET strategy yielded 12.71 QALYs compared with 11.11 QALYs for ET, resulting in ICERs of $19,452/LY and $17,448/QALY (Additional file 1: Table S5).

## Discussion

The approval of ABE as the first CDK4/6 inhibitor for eBC treatment in China marked a significant milestone in HR + /HER2− adjuvant therapy. The Chinese Society of Clinical Oncology has listed ABE in combination with ET as a recommended treatment option for eBC [35]. Although its effectiveness and safety have been widely recognized by clinical experts, its cost-effectiveness in clinical practice has yet to be confirmed. To the best of our knowledge, this study is the first to compare the economic evaluation of ABE + ET and ET alone as an adjunctive therapy for HR+/HER2− eBC in China.

We compared the cost-effectiveness of ABE + ET and ET alone, adopting the perspective of the Chinese healthcare system. Our cost analysis adopted the latest prices from medical insurance negotiations and adjusts prices from other relevant sources in China. The results estimated the ICER of ABE + ET treatment to be $22,385/QALY in standard parametric model, $15,264/QALY in mixture cure model and $12,191/QALY in non-mixture cure model. When using a threshold of three times the GDP per capita, the ABE + ET group was cost-effective compared to the ET group. However, when using a threshold of one time the GDP per capita, the ET group was considered cost-effective over the ABE + ET group. The one-way sensitivity analysis identified outcome discount rate and health state utility of iDFS as the primary drivers in the model. The results in probabilistic sensitivity analysis further reinforce the base-case results. Additionally, the subgroup analysis found that, among patients with Ki-67 ≥ 20%, the ICER of ABE + ET did not meet 3 GDP per capita per QALY, despite providing a greater incremental cost compared to the base case.

We used time horizons that were long enough to capture all the clinical outcomes and costs associated to the disease and the treatment. In the base-case analysis, this statement translates into a lifetime time horizon (until less than 1% of patients are still at risk), resulting in time horizons of 52 years for the ABE + ET group and 51 years for the ET group in the context of MonarchE. Furthermore, we considered the impact of simulating outcomes up to the average life expectancy, resulting in a 26-year time horizon for the scenario analysis. By shortening the time horizon of the study, the incremental QALY decreased, while the incremental cost did not exhibit significant changes. As a result, the ICERs increased.

In most cancer drug assessments, survival curves are used as the basis for calculating expected LYs and QALYs [32, 36]. Due to the limited study timeframe, a significant portion of clinical and economic benefits will occur outside the curve, making appropriate extrapolation methods crucial in capturing the value of treatment plans [37]. This study used standard parameter and cure model extrapolation curves. The latter demonstrated greater flexibility in fitting follow-up periods, and patients in the cure model had higher long-term survival rates than those in the standard parameter model. In a Markov model, the calculation of transition probabilities requires the derivation of the number of individuals in each state from the iDFS curve. As transition probabilities are obtained from the decline in the curve and the study adjusts the curve additionally, differences in transition probabilities do not necessarily equal differences in extrapolated iDFS rates. The results obtained using three different extrapolation methods showed some differences, but they did not alter the conclusion.

The economic burden of breast cancer on both patients and the healthcare system is substantial. Breast cancer costs are estimated to account for 9.9% of all cancer-related healthcare costs, with drugs being the largest contributor [38]. Additionally, it is noted that early detection and treatment can significantly reduce this financial strain [39]. This study provides evidence that the use of ABE + ET results in additional benefits for patients with HR+/HER2− eBC and is economically viable according to the available WTP thresholds. These findings could influence Chinese policy makers in their decision to include ABE on the national reimbursement drug list. The model results could be further improved by considering societal perspectives, including the impact of patients' lost productivity and caregiver burden. The PURPOSE study performed in the UK found that patients with eBC were less likely to be unemployed compared to those with metastatic breast cancer [40], which could have a positive economic impact from a societal perspective.

Several limitations also need to be recognized in our study. Firstly, the monarchE trial did not provide information on medication regimens after relapse and there are numerous options available in real-world settings. Due to the lack of relevant literature or databases, we relied on clinical expertise to define the usage of certain resources and estimate associated costs, which may lead to underestimation or overestimation of costs. Furthermore, patients in the MonarchE trial were mostly from North America and Europe, with only 23.8% Asian, which introduce uncertainty to the results. Additionally, a suitable utility value for the target patient population based on Chinese patients was not available, therefore utility values from other countries were used in the analysis. Differences in geography and culture between countries may impact utility weights. However, a sensitivity analysis was performed, and it showed that the health utility value does not have a significant effect on the stability of the model. Moreover, due to the absence of registry data, we relied solely on parametric models for extrapolation. This reliance on parametric models was a significant source of uncertainty in our results.

## Conclusion

From the perspective of Chinese healthcare system, the adjuvant treatment of ABE + ET might not have an economic advantage over ET at a WTP threshold of $11,864/QALY (1 GDP per capita). However, ABE + ET was likely to be cost-effective at a WTP threshold of $35,594/QALY (3 GDP per capita). Due to the limited iDFS maturity, we used standard parameter and cure model models to capture the value of treatment plans. Although the results obtained using three different extrapolation methods showed some differences, they did not change the overall conclusion. Further analysis will be conducted once data from longer-term studies become available.

### Supplementary Information

**Additional file 1: Figure S1.** A scatterplot in the cost-effectiveness plane. **Table S1.** Results of fitting to the observed data in ITT population. **Table S2.** Best fitting and the value of the parameter in ITT population. **Table S3.** Results of fitting to the observed data in Ki-67 ≥ 20% population. **Table S4.** Best fitting and the value of the parameter in Ki-67 ≥ 20% population. **Table S5.** Subgroup analysis results.

## Data Availability

The detailed information of parameters and their sources were performed in the tables and appendixes.
