# Supplementary material for: Cost-effectiveness of abemaciclib plus endocrine therapy in high-risk HR+/HER2–early breast cancer in China
Source: Cost Eff Resour Alloc. 2023 Nov 27;21:91. doi: 10.1186/s12962-023-00499-9 (PMC10683222; doi:10.1186/s12962-023-00499-9)
Supplement: Supplementary file 1 — Additional file 1: Figure S1. A scatterplot in the cost-effectiveness plane. Table S1. Results of fitting to the observed data in ITT population. Table S2. Best fitting and the value of the parameter in ITT population. Table S3. Results of fitting to the observed data in Ki-67 ≥ 20% population. Table S4. Best fitting and the value of the parameter in Ki-67 ≥ 20% population. Table S5. Subgroup analysis results. [file 12962_2023_499_MOESM1_ESM.docx]

**Cost-effectiveness of Abemaciclib plus Endocrine Therapy in High-risk HR+/HER2– Early Breast Cancer in China**

*Qiran Wei, BEc^1,2^, YuTing Xu, BS^1,2^, Wei Liu, BS^1,2^, Xin Guan, PhD^1,2*^*

^1^School of International Pharmaceutical Business, China Pharmaceutical University, Nanjing 211198, Jiangsu Province, China;

^2^Center for Pharmacoeconomics and Outcomes Research of China Pharmaceutical University, Nanjing 211198, Jiangsu Province, China;

**Correspondence to:**

*Corresponding author: Xin Guan

E-mail: guanxin@cpu.edu.cn

Phone number: +86 15251751153

**
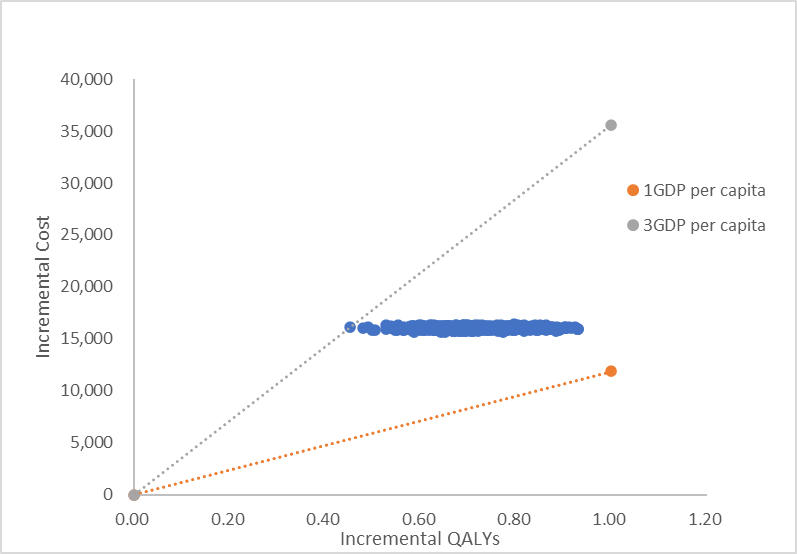
**

**Figure S1 A scatterplot in the cost-effectiveness plane** QALY: quality-adjusted life-years.

**Table S1 Results of fitting to the observed data in ITT population**

|  | ABE +ET | ET |
| --- | --- | --- |
|  | AIC | AIC |
| Expontional | 3146.555 | 4197.151 |
| Gamma | 3141.277 | 4177.396 |
| Gompertz | 3146.638 | 4185.252 |
| Weibull | 3141.764 | 4177.900 |
| Log-Logistic | 3140.964 | 4177.531 |
| Log-Normal | 3132.606 | 4175.018 |
| mixe | 3112.095 | 4150.599 |
| mixgam | 3103.863 | 4163.087 |
| mixgom | 3111.735 | 4143.885 |
| mixw | 3105.113 | 4143.413 |
| mixll | 3104.508 | 4143.831 |
| mixln | 3098.103 | 3098.103 |
| nmixe | 3112.104 | 4163.108 |
| nmixgam | 3103.847 | 4143.52 |
| nmixgom | 3111.730 | 4150.572 |
| nmixw | 3105.005 | 4143.839 |
| nmixll | 3104.518 | 4143.801 |
| nmixln | 3098.374 | 4141.124 |

ABE + ET, abemaciclib + endocrine therapy; ET, endocrine therapy; iDFS, invasive disease-free survival; AIC: Akaike information criterion.

**Table S2 Best fitting and the value of the parameter in ITT population**

|  | Model | Best fitting | P_cure_ | $\mu$ | $\sigma$ |
| --- | --- | --- | --- | --- | --- |
| ABE + ET iDFS | Standard | Lognormal ($\mu$, $\sigma$) | / | 5.837 | 1.836 |
|  | Mixture | Lognormal ($\mu$, $\sigma$) | 0.657 | 4.387 | 1.452 |
|  | Un-mixture | Lognormal ($\mu$, $\sigma$) | 0.665 | 4.555 | 1.483 |
| ET iDFS | Standard | Lognormal ($\mu$, $\sigma$) | / | 5.245 | 1.630 |
|  | Mixture | Lognormal ($\mu$, $\sigma$) | 0.000 | 5.300 | 1.630 |
|  | Un-mixture | Lognormal ($\mu$, $\sigma$) | 0.000 | 8.229 | 2.195 |

ABE + ET, abemaciclib + endocrine therapy; ET, endocrine therapy; iDFS, invasive disease-free survival.

**Table S3 Results of fitting to the observed data in Ki-67 ≥ 20% population**

|  |  | Exponential | Gamma | Gompertz | Weibull | Loglogistic | Lognormal |
| --- | --- | --- | --- | --- | --- | --- | --- |
| ABE + ET iDFS | AIC | 1598.033 | 1600.004 | 1599.284 | 1599.985 | 1600.527 | 1605.427 |
| ET iDFS | AIC | 2183.490 | 2176.382 | 2178.451 | 2176.331 | 2176.434 | 2181.136 |

ABE + ET, abemaciclib + endocrine therapy; ET, endocrine therapy; iDFS, invasive disease-free survival; AIC: Akaike information criterion.

**Table S4 Best fitting and the value of the parameter in Ki-67 ≥ 20% population**

|  | Best fitting | $\lambda$ | $\gamma$ |
| --- | --- | --- | --- |
| ABE + ET iDFS | Expontional ($\lambda$) | 0.003715736 | / |
| ET iDFS | Weibull ($\lambda$, $\gamma$) | 1.2540058 | 123.7090071 |

ABE + ET, abemaciclib + endocrine therapy; ET, endocrine therapy; iDFS, invasive disease-free survival.

**Table S5 Subgroup analysis results**

| Results | ABE + ET | ET | Difference |
| --- | --- | --- | --- |
| LYs | 13.44 | 12.00 | 1.44 |
| QALYs | 12.71 | 11.11 | 1.60 |
| Total cost ($) | 44,586.45 | 16,709.21 | 27,877.23 |
| ICER ($/LY) | 19,452 |  |  |
| ICER ($/QALY) | 17,448 |  |  |

ABE + ET, abemaciclib + endocrine therapy; ET, endocrine therapy; LY, life-year; QALY: quality-adjusted life-year; ICER: incremental cost-effectiveness ratio.
